# Establishing Australia’s national linked COVID Register

**DOI:** 10.23889/ijpds.v9i5.2806

**Published:** 2024-09-18

**Authors:** Frederieke Petrović-van der Deen, Sheree Gibb, Melissa McLeod, Andrea Teng

**Affiliations:** 1 University of Otago

## Objectives and Approach

This study examined the impact of two sources of bias on differences in ethnic disparities in non-communicable disease rates in New Zealand (NZ). Data were sourced from Stats NZ’s Integrated Data Infrastructure (IDI), a collection of deidentified whole-population administrative (eg, health, justice, housing) and survey datasets (eg, NZ Census) linked at the individual level using probabilistic linkage procedures. Linking several datasets reduces the size of the population available for study because not all individuals can be linked. In addition, there are several sources of ethnicity information that may disagree with each other. We will illustrate the impact of population loss due to linkage and ethnicity data source on Māori-European gaps in lung cancer and cardiovascular disease.

## Results

Our results showed that the choice of ethnicity information source and the population used had an impact on the size of ethnic disparities in lung cancer and cardiovascular disease. For lung cancer the age standardised rate ratio for Māori:European ranged from 2.88 to 3.21, and for CVD 1.70 to 1.87. Population loss and ethnicity data source each had independent effects on the size of ethnic differences.

## Conclusions

Different combinations of population and ethnicity information source produced different estimates of ethnic gaps in lung cancer and CVD prevalence. Population and source of ethnicity data both had independent effects on the size of ethnic differences.

## Implications

Comparisons of ethnic differences in disease prevalence between studies, or over time, may be misleading if they do not use the same population and ethnicity data source.

